# Pancharatnam–Berry phase in condensate of indirect excitons

**DOI:** 10.1038/s41467-018-04667-x

**Published:** 2018-06-04

**Authors:** J. R. Leonard, A. A. High, A. T. Hammack, M. M. Fogler, L. V. Butov, K. L. Campman, A. C. Gossard

**Affiliations:** 1 Department of Physics, University of California at San Diego, La Jolla, CA 92093-0319 USA; 2 Materials Department, University of California at Santa Barbara, Santa Barbara, CA 93106-5050 USA

## Abstract

The Pancharatnam–Berry phase is a geometric phase acquired over a cycle of parameters in the Hamiltonian governing the evolution of the system. Here, we report on the observation of the Pancharatnam–Berry phase in a condensate of indirect excitons (IXs) in a GaAs-coupled quantum well structure. The Pancharatnam–Berry phase is directly measured by detecting phase shifts of interference fringes in IX interference patterns. Correlations are found between the phase shifts, polarization pattern of IX emission, and onset of IX spontaneous coherence. The evolving Pancharatnam–Berry phase is acquired due to coherent spin precession in IX condensate and is observed with no decay over lengths exceeding 10 μm indicating long-range coherent spin transport.

## Abstract (web summary)

Geometric phases in matter are of fundamental interest. Here the authors observe the Pancharatnam–Berry phase in matter waves by imaging light interference patterns from cold gases of indirect excitons and find long-range coherent spin transport in the system.

## Introduction

The Pancharatnam–Berry phase was discovered by Pancharatnam in studies of polarized light^1^ and introduced by Berry as a topological phase for matter wave functions^2^. For light, the Pancharatnam–Berry phase is measured in laser interferometers^3,4^ and exploited in optical elements^5,6^. Excitons are matter waves that directly transform to photons inheriting their coherence and polarization. This makes excitons a unique interface between matter and light and a unique system for exploring the Pancharatnam–Berry phase for matter waves by light interference experiments.

Recent studies led to the discovery of polarization textures in light emission of indirect excitons (IXs)^7–16^ and exciton–polaritons^17–19^. The Pancharatnam–Berry phase appears when the polarization state of light changes^1^. This connection of the Pancharatnam–Berry phase to polarization makes it an intrinsic phenomenon for polarization textures.

An IX is a bound pair of an electron and a hole confined in spatially separated layers. IXs are realized in coupled quantum well (CQW) structures. Due to their long lifetimes IXs can cool below the temperature of quantum degeneracy and form a condensate in momentum (*k*) space^7^. IX condensation is detected by measurement of IX spontaneous coherence with a coherence length much larger than in a classical gas^7^. The large coherence length observed in an IX condensate, reaching ~10 μm, indicates coherent IX transport with suppressed scattering^7^, in agreement with theory^20^.

A cold IX gas is realized in the regions of the external ring and localized bright spot (LBS) rings in the IX emission^7,8^. These rings form on the boundaries of electron-rich and hole-rich regions created by current through the structure and optical excitation, respectively; see ref. ^21^ and references therein. An LBS is a stable, well defined, and tunable source of cold IXs^21^, thus an ideal system for studying coherence and polarization phenomena. Different LBS offer IX sources of different strength and spatial extension; furthermore, these parameters can be controlled by optical excitation and voltage^21^. This variability gives the opportunity to measure correlations between coherence and polarization. Here, we explore LBS to uncover the Pancharatnam–Berry phase in a condensate of IXs.

## Results

### Experiment

**Fig. 1:**
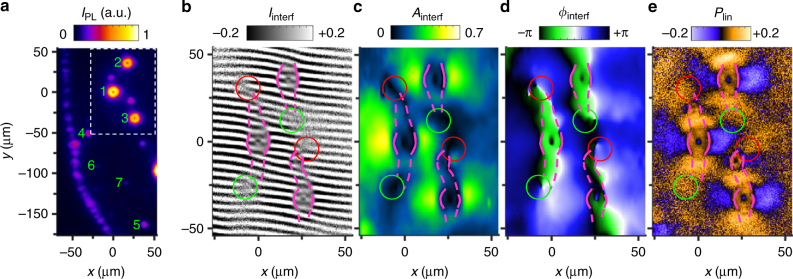
IX coherence and polarization patterns. **a** IX emission image showing LBS 1–7 numbered according to their emission power. The external ring is seen on the left. **b**–**e** Coherence and polarization patterns in the region of LBS 1–3 marked by the dashed rectangle in **a**. **b** Shift-interference pattern of IX emission, *I*_interf_(*x*, *y*). The shift *δx* = 2 μm. **c**, **d** Amplitude (**c**) and phase (**d**) of interference fringes in **b**, *A*_interf_(*x*, *y*) and *ϕ*_interf_(*x*, *y*). **e** The linear polarization of IX emission, *P*_linear_(*x*, *y*). In **b**, the positions of phase shifts of interference fringes are marked by magenta lines and the positions of left (right) forks of interference fringes are marked by green (red) circles. The lines are solid in the circular region around each LBS where the phase shifts are sharp and dashed outside these regions where the phase shifts are smoother. These lines and circles are copied to **c**–**e** to show spatial correlations in *A*_interf_(*x*, *y*), *ϕ*_interf_(*x*, *y*), and *P*_linear_(*x*, *y*). Excitation power *P* = 1.2 mW. *T*_bath_ = 0.1 K

Figure 1b shows the interference pattern of IX emission measured by shift-interferometry: The emission images produced by each of the two arms of the Mach–Zehnder interferometer are shifted with respect to each other to measure the interference between the emission of IXs separated by *δ****r*** in the CQW plane (Supplementary Note 3).

Spontaneous coherence of matter waves is equivalent to condensation of particles in *k*-space. The Fourier transform of the first-order coherence function *g*_1_(*δx*) gives the particle distribution *n*_*k*_. The width of *g*_1_(*δx*), the coherence length *ξ*, is inversely proportional to the width of *n*_*k*_. In a classical gas, *ξ* is close to the thermal de Broglie wavelength *λ*_dB_ = (2*πħ*^2^/*mT*)^1/2^ and is small (*λ*_dB_ ~ 0.5 μm for IXs with *m* = 0.22*m*_0_ at *T* = 0.1 K). The measurement of spontaneous coherence with $\xi \gg \lambda _{{\mathrm{dB}}}$ is a direct measurement of Bose–Einstein condensation.

The measured amplitude of interference fringes *A*_interf_(*δx*) is given by the convolution of *g*_1_(*δx*) with the point-spread function (PSF) of the optical system^22^. The PSF width corresponds to the spatial resolution (~1.5 μm in this experiment). For a classical IX gas, *g*_1_(*δx*) is narrow and *A*_interf_(*δx*) fits well to the PSF, while for the IX condensate, *g*_1_(*δx*) and, in turn, *A*_interf_(*δx*) extend to large *δx*^7^. While a more detailed picture is obtained by measuring *g*_1_(*δx*) as in ref. ^7^, mapping IX condensate can be done by measuring *A*_interf_(*x*, *y*) at one value of *δx* chosen to exceed both *λ*_dB_ and the PSF width. For such *δx*, a low *A*_interf_ is observed for a classical gas and a high *A*_interf_ for the condensate. For the parameters of our system, *δx* = 2 μm is optimal for this experiment.

The IX gas is classical close to the heating sources in the LBS central region (this heating is due to the current filament at the LBS centre and the binding energy released at IX formation^21^). This is revealed by the small amplitude of the interference fringes *A*_interf_ at *r* < *r*_coh_ (Fig. 1c). Away from the heating sources, IXs cool down and approach the condensation temperature. At *r* = *r*_coh_, *A*_interf_ sharply rises, indicating the condensation.

Figure 1b, d shows that the phase of interference fringes sharply changes at the distance from the LBS centre *r* = *r*_phase_. The comparison of Fig. 1b–d shows that the phase shifts occur at the same location as condensation, *r*_phase_ = *r*_coh_.

The phase shifts are sharp in the circular region around each LBS. However smoother phase shifts can be followed further (as shown by dashed magenta lines in Fig. 1b). The lines embracing the phase domains of interference fringes end by left- and right-forks of interference fringes at the opposite ends of the domain (the forks are shown by circles in Fig. 1b), indicating that the forks originate from the phase domains.

To uncover the origin of the phase shifts and associated phase domains of interference fringes, we compare their locations with the pattern of linear polarization of IX emission (Fig. 1e). A ring of linear polarization is seen for each LBS in the region *r* < *r*_linear_ where the IX gas is classical, *r*_linear_ = *r*_coh_ (compare Fig. 1c, e). This linear polarization originates from the distribution of IXs over the linearly polarized IX states^8^. A helical IX polarization texture winding by 2*π* around the origin, i.e. a vortex of linear polarization, emerges at *r* > *r*_linear_ where the IX condensate forms (Fig. 1e).

The comparison of Fig. 1b–e shows that for all LBS sources, the phase shifts of interference fringes are observed when the polarization state of IX emission sharply changes. To examine this relationship, we measured IX coherence and polarization patterns at different laser exciation powers *P*. As in ref. ^21^, we also adjusted the applied voltage *V* keeping the external ring radius constant. The simultaneous increase of *P* and *V* leads to the enhancement of both electron and hole sources and, as a result, the exciton source at each LBS.

**Fig. 2:**
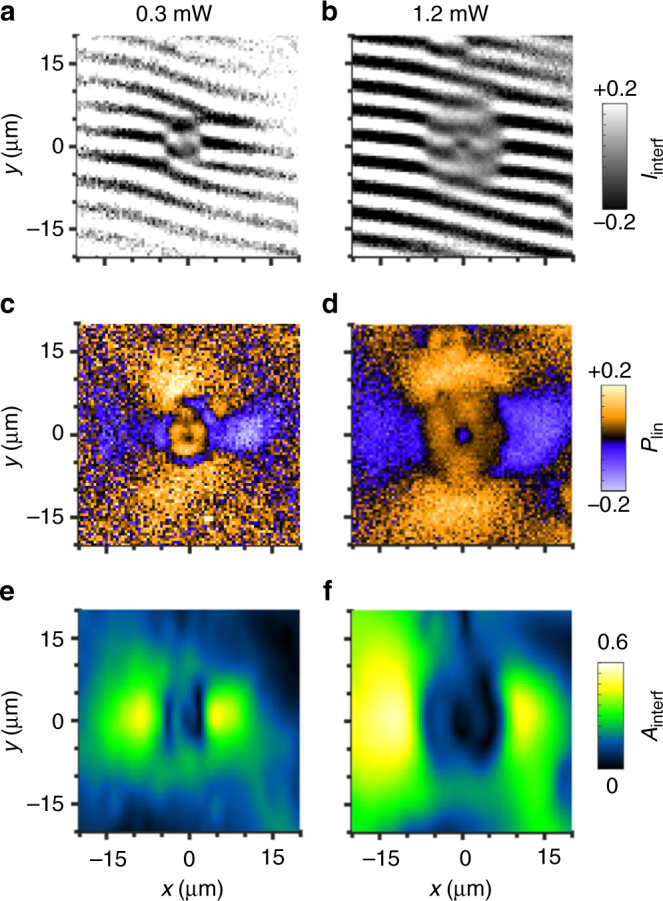
IX coherence and polarization patterns vs power. **a**, **b** Shift-interference pattern of IX emission. *δx* = 2 μm. **c**, **d** The linear polarization of IX emission. **e**, **f** Amplitude of interference fringes in **a**, **b**. *P* = 0.3 (1.2) mW for left (right). *T*_bath_ = 0.1 K. The data correspond to LBS 1

Increasing the excitation power increases *r*_phase_ (Fig. 2a, b), *r*_linear_ (Fig. 2c, d), and *r*_coh_ (Fig. 2e, f). The increase of *r*_coh_ with *P* follows an enhanced heating at the LBS central region due to the enhanced electron and hole sources. Figure 2 shows that *r*_linear_ stays equal to *r*_coh_ with increasing *P*, confirming that the polarization textures appear in the IX condensate, in agreement with theory^7,8^. Remarkably, Fig. 2 shows that *r*_phase_ also keeps equal to *r*_linear_ with increasing *P*.

**Fig. 3:**
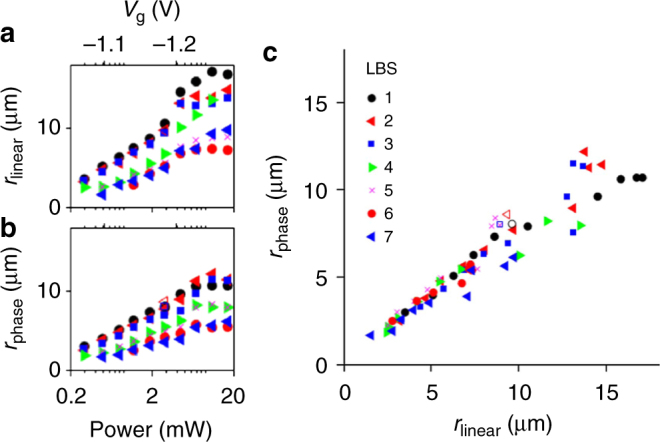
Correlation between the phase shifts and polarization pattern. **a** The radius at which linear polarization changes sign, *r*_linear_ as a function of laser power, *P* for different LBS. **b** The radius at which the phase of interference fringes shifts, *r*_phase_ as a function of laser power, *P* for different LBS. **c** *r*_phase_ vs *r*_linear_. Solid (open) symbols correspond to 2 µm shift in $\hat x$ ($\hat y$). *T*_bath_ = 0.1 K. The data for different LBS and different *P* collapse on a universal line *r*_phase_ ≈ *r*_linear_

We found a universal relationship between *r*_phase_ and *r*_linear_ measured for many LBS over a broad range of *P*. Large variations of both *r*_phase_ and *r*_linear_ are observed for different LBS and *P* (Fig. 3a, b). However, all data collapse on a universal line *r*_phase_ = *r*_linear_ (Fig. 3c). Slight deviation of the slope of the line *r*_phase_(*r*_linear_) from 1 may be related to the calibration accuracy (Supplementary Note 3).

**Fig. 4:**
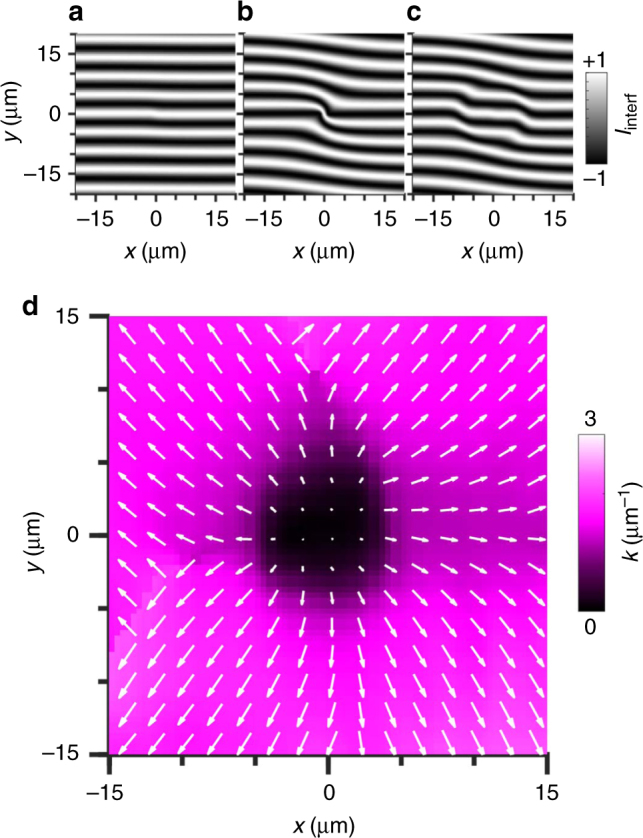
Spatial pattern of IX momentum **k**. **a**–**c** Simulation of IX shift-interference pattern for *k* = 0.1 μm^−1^ (**a**), for *k* = 1.5 μm^−1^ (**b**), and for *k* = 0.1 μm^−1^ at *r* < *r*_*k*_ and *k* = 1.5 μm^−1^ at *r* > *r*_k_ (**c**). *r*_*k*_ = 8 μm. *δx* = 2 μm. IXs propagate away from the origin. **d** Pattern of *k* extracted from shift-interferometry measurements with 2 μm shift in $\hat x$ and $\hat y$. The direction and size of arrows indicate **k** direction and magnitude, respectively. *P* = 2.9 mW. *T*_bath_ = 0.1 K. The data correspond to LBS 6

The phase of IX wave function *ψ*(**r**) acquired as IXs propagate from the origin can be derived from the interference pattern simulated using *I*_interf_(**r**) = $\left| {\psi ({\bf{r}} - \delta {\bf{r}}{\mathrm{/}}2) + {\mathrm {e}}^{{\mathrm {i}}q_ty}\psi ({\bf{r}} + \delta {\bf{r}}{\mathrm{/}}2)} \right|^2$, where *q*_*t*_ = 2*πα*/*λ* sets the period of the interference fringes, *α* is a small tilt angle between the image planes of the interferometer arms, and *λ* is the emission wavelength. Figure 4a–c show the simulated *I*_interf_(*x*, *y*) for *ψ*(**r**) = e^i**k**·**r**^ and the shift *δx* = 2 μm for IXs radially propagating from the origin with a small *k* (Fig. 4a), with a larger *k* (Fig. 4b), and with the small *k* at *r* < *r*_*k*_ and larger *k* at *r* > *r*_*k*_ (Fig. 4c). For *k* = 0, the interference fringes are parallel lines separated by *D* = *λ*/*α*. A deviation of the *N*th fringe from its zero-*k* position is given by *δy*_*N*_ = −*D*/(2*π*)·*k*_*x*_*δx*. Phase shifts of interference fringes, i.e., jumps in *δy*_*N*_, correspond to jumps in *k* (Fig. 4c). The values of *k* at *r* < *r*_k_ and *r* > *r*_k_ in Fig. 4c were selected to qualitatively reproduce the measured *I*_interf_ in Fig. 1 and illustrate a jump in *k* at *r* = *r*_phase_.

The *x* and *y* components of **k** were derived by fitting the patterns of interference fringes measured for shifts in the *x* and *y* directions using *k*_*x*_(*x*, *y*) = −2*π*/*D* · *δy*_*N*_(*x*, *y*)/*δx* and *k*_*y*_(*x*, *y*) = −2*π*/*D* · *δy*_*N*_(*x*, *y*)/*δy*, respectively. The positions of faint interference fringes at *r* < *r*_coh_ and pronounced interference fringes at *r* > *r*_coh_ were used in the estimate of **k**. This allowed obtaining a map of IX momentum **k** describing the evolving IX phase (Fig. 4d). This map shows that IXs propagate away from the LBS source and sharply acquire an additional evolving phase *ϕ* = *kr* at *r* = *r*_phase_. This phase is revealed in Fig. 4d by a jump in associated momentum *k*.

The experiment shows that the phase shifts correlate with the polarization pattern of IX emission and onset of IX spontaneous coherence. The correlation between the phase shift and the polarization change identifies the phase as the Pancharatnam–Berry phase acquired in a condensate of IXs. This phenomenon is discussed below.

The spatial separation of an electron and a hole in an IX reduces the overlap of the electron and hole wave functions suppressing the spin relaxation mechanism due to electron–hole exchange^23^. In a classical IX gas, spin transport in the studied structure is limited by 1−2 μm^24^ due to Dyakonov–Perel spin relaxation^25^. As a result, for uncondensed IXs at *r* < *r*_coh_, the spin relaxation is fast and coherent spin precession is not observed. However, the suppression of scattering in IX condensate results in the suppression of the Dyakonov–Perel and Elliott–Yafet mechanisms of spin relaxation^25^ enabling long-range coherent spin transport in IX condensate. Therefore, IX condensation at *r* > *r*_coh_ dramatically enhances the spin relaxation time leading to coherent spin precession and, in turn, precession of the polarization state of IX emission. This precession generates the evolving Pancharatnam–Berry phase of IXs, which is detected as the shift of interference fringes. Figure 4d shows that no decay of the evolving Pancharatnam–Berry phase is observed over macroscopic lengths exceeding 10 μm. This indicates the achievement of macroscopic long-range coherent spin transport in the IX condensate.

### Simulation

To demonstrate a concept of the Pancharatnam–Berry phase acquired due to the IX spin precession, we simulate the polarization evolution within the model of IX spin precession^8^ (a brief description is given in Supplementary Note 4). We use the electron and hole spin–orbit interaction constants and splittings between four IX states (with spin projections *J*_*z*_ = ±2, ±1) obtained to fit the IX polarization patterns^8^. The initial polarization in the simulations is taken as horizontal to follow the experiment (Fig. 1e).

**Fig. 5:**
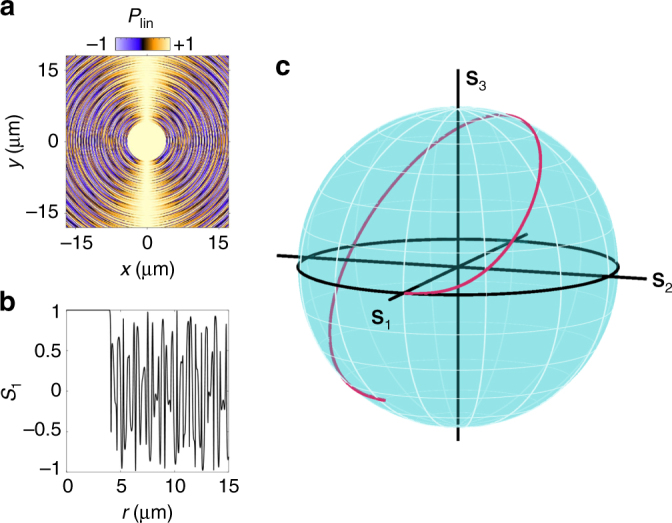
Simulation of IX polarization state. **a** Simulated S1 Stokes’ vector component corresponding to linear polarization of IX emission. Initial conditions at *r* = *r*_p_ correspond to linear polarization. Oscillatory S1 behaviour due to coherent spin precession occurs for *r* > *r*_p_. *r*_p_ = 4 μm. **b** Diagonal cross-section of **a**. **c** IX polarization state on Poincaré sphere for one fast polarization oscillation cycle in **a**

The simulated S1 component of Stokes’ vector corresponding to linear polarization of IX emission is presented in Fig. 5a, b. The polarization shows an oscillatory behaviour. Its long-scale component is responsible for the polarization pattern shown in Fig. 1e and studied earlier in ref. ^8^. The short-scale component has the spatial period ~0.3 μm and is not resolved with 1.5 μm optical resolution in the imaging experiment. However, these fast changes of the polarization state generate the evolving Pancharatnam–Berry phase of IXs. Figure 5c shows the simulated IX polarization state on the Poincaré sphere for one fast polarization oscillation cycle in Fig. 5a. The IX polarization state goes over a nearly closed contour on the Poincaré sphere. The Pancharatnam–Berry phase acquired by IXs over this contour can be estimated by connecting the initial and final points and calculating half the solid angle subtended by the obtained contour at the centre of the sphere, *Ω*/2 (ref. ^1^). In turn, a momentum *k*_PB_ associated with the acquired Pancharatnam–Berry phase can be estimated as ~*Ω*/(2*l*) where *l* is the IX path passed during the polarization cycle. For *Ω*/2 ~ *π*/2 (Fig. 5c) and *l* ~ 0.3 μm (Fig. 5a, b), this gives *k*_PB_ ~ 5 μm^−1^, in qualitative agreement with the jump in IX momentum (Fig. 4d), which occurs when the coherent spin precession generating the evolving Pancharatnam–Berry phase starts in IX condensate.

In summary, shift-interferometry and polarization imaging show that the phase shifts of interference fringes correlate with the polarization pattern of IX emission and onset of IX spontaneous coherence, demonstrating the Pancharatnam–Berry phase in a condensate of IXs. The measured Pancharatnam–Berry phase indicates long-range coherent spin transport.

## Methods

The experiments are performed on *n* − *i* − *n* GaAs/AlGaAs CQW structure. The *i* region consists of a single pair of 8-nm GaAs QWs separated by a 4-nm Al_0.33_Ga_0.67_As barrier and surrounded by 200-nm Al_0.33_Ga_0.67_As layers. The *n* layers are Si-doped GaAs with Si concentration 5 × 10^17^ cm^−3^. The indirect regime where IXs form the ground state is realized by the voltage applied between *n* layers. The small in-plane disorder in the CQW is indicated by the emission linewidth of 1 meV. IXs cool to temperatures within ~50 mK of the lattice temperature^26^, which was lowered to 100 mK in an optical dilution refrigerator. This cools IXs well below the temperature of quantum degeneracy, which is in the range of a few kelvin for typical IX density 10^10^ cm^−2^(ref. ^26^). The laser excitation is performed by a 633 nm HeNe laser. It is more than 400 meV above the energy of IXs and farther than 80 μm away from the studied region. Therefore, IX coherence and polarization are not induced by photoexcitation and form spontaneously. LBS are sources of cold IXs due to their separation from the laser excitation spot.

### Data availability

All relevant data are available from the authors.

## Electronic supplementary material

Supplementary Information
