# Supplementary material for: Pancharatnam–Berry phase in condensate of indirect excitons
Source: Nat Commun. 2018 Jun 4;9:2158. doi: 10.1038/s41467-018-04667-x (PMC5986757; doi:10.1038/s41467-018-04667-x)
Supplement: Supplementary file 1 — Supplementary Information [file 41467_2018_4667_MOESM1_ESM.pdf]

Supplementary Information
Pancharatnam-Berry phase in condensate of indirect excitons

J. R. Leonard, A. A. High, A. T. Hammack, M. M. Fogler, L. V. Butov
Department of Physics, University of California at San Diego, La Jolla, California 92093-0319, USA

K. L. Campman, A. C. Gossard
Materials Department, University of California at Santa Barbara, Santa Barbara, California 93106-5050, USA

Supplementary Note 1: Coupled Quantum Well structure

Supplementary Figure 1 shows the energy diagram of the coupled quantum well (CQW) structure.

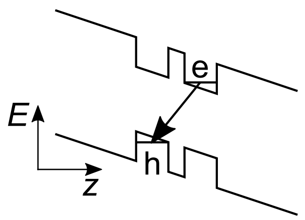

Supplementary Figure 1: **IX band diagram.** Energy diagram showing CQW structure with an applied electric field along the z-axis. e (h) indicate an electron (hole). The arrow indicates an indirect exciton.

Supplementary Note 2: Supplemental Data

The lower row in Supplementary Figure 2 is similar to Figure 1 in the main text, the upper row of Supplementary Figure 2 shows the same raw data without markup.

Supplementary Note 3: Experimental Setup

We use a Mach-Zehnder (MZ) interferometer to probe the coherence of the exciton system (Supplementary Figure 3). The emission beam is made parallel by an objective inside the optical dilution refrigerator and lenses. A combination of a quarter-wave plate and a half-wave plate converts the measured polarization of the emission to the y-polarization, which is then selected by a linear polarizer. This ensures only y-polarized light enters the MZ interferometer eliminating polarization-dependent effects in the interferometer and spectrometer. The emission is split between arms of the MZ interferometer. The path lengths of the arms are equal. The interfering emission images produced by arm 1 and 2 of the MZ interferometer are shifted relative to each other along x (or y) directions to measure the interference between the emission of excitons, which are laterally separated by δx (or δy). After the interferometer, the emission is filtered by an interference filter of linewidth ±5 nm adjusted to the exciton emission wavelength ≈ 800nm. The filtered signal is focused to produce an image, which is measured by a liquid-nitrogen cooled CCD. We measure exciton emission intensity I_1 for arm 1 open, intensity I_2 for arm 2 open, and intensity I_{12} for both arms open, and then calculate

$$I_{\mathrm{interf}} = \frac{I_{12} - I_1 - I_2}{2\sqrt{I_1 I_2}} \tag{1}$$

shown in Supplementary Figure 2a. In general, for two partially coherent sources located at $\mathbf{r_1}$ and $\mathbf{r_2}$, one has the relation [1],

$$I_{\mathrm{interf}} = \cos\delta\theta\,(\mathbf{r}_1, \mathbf{r}_2)\,\zeta\,(\mathbf{r}_1, \mathbf{r}_2)\,, \tag{2}$$

where $\delta\theta\,(\mathbf{r}_1, \mathbf{r}_2)$ is the phase difference of the two sources and $\zeta\,(\mathbf{r}_1, \mathbf{r}_2)$ is their degree of coherence. In our experimental geometry, there is a small tilt angle α between the image planes of the two arms. As a result, the phase difference

$$\delta\theta\,(\mathbf{r}_1, \mathbf{r}_2) = q_y y + \phi\,(\mathbf{r}_1, \mathbf{r}_2) \tag{3}$$

has a component linear in y - the coordinate in the direction perpendicular to the tilt axis - which produces periodic oscillation of I_{interf}. The period of the interference fringes is set by $q_y = 2\pi\alpha/\lambda$. The coherence function $\zeta\,(\mathbf{r}_1, \mathbf{r}_2)$ for $\mathbf{r}_1 - \mathbf{r}_2 = \delta\mathbf{r}$ is given by the amplitude of these interference fringes. The amplitude and phase of the interference fringes in Supplemental Figure 2 (b) and (c) were calculated from the interference pattern (Supplementary Figure 2(a)) using a two dimensional windowed Fourier transform with a gaussian window.

At high temperatures, the linear polarization $P_{\mathrm{linear}} = \left(I_x - I_y\right) / \left(I_x + I_y\right)$ of exciton emission is expected to vanish, and indeed for $T_{\mathrm{bath}} = 7$ K, in the region of an LBS, P_{linear} is small, $\lesssim 5\%$, that is within the polarization calibration accuracy. r_{linear} is defined as the radius where P_{linear} along $\hat{x}$ changes sign. The slope of the line $r_{\mathrm{phase}}(r_{\mathrm{linear}})$ (Fig. 3c in the main text) is equal to 1 within the calibration accuracy.

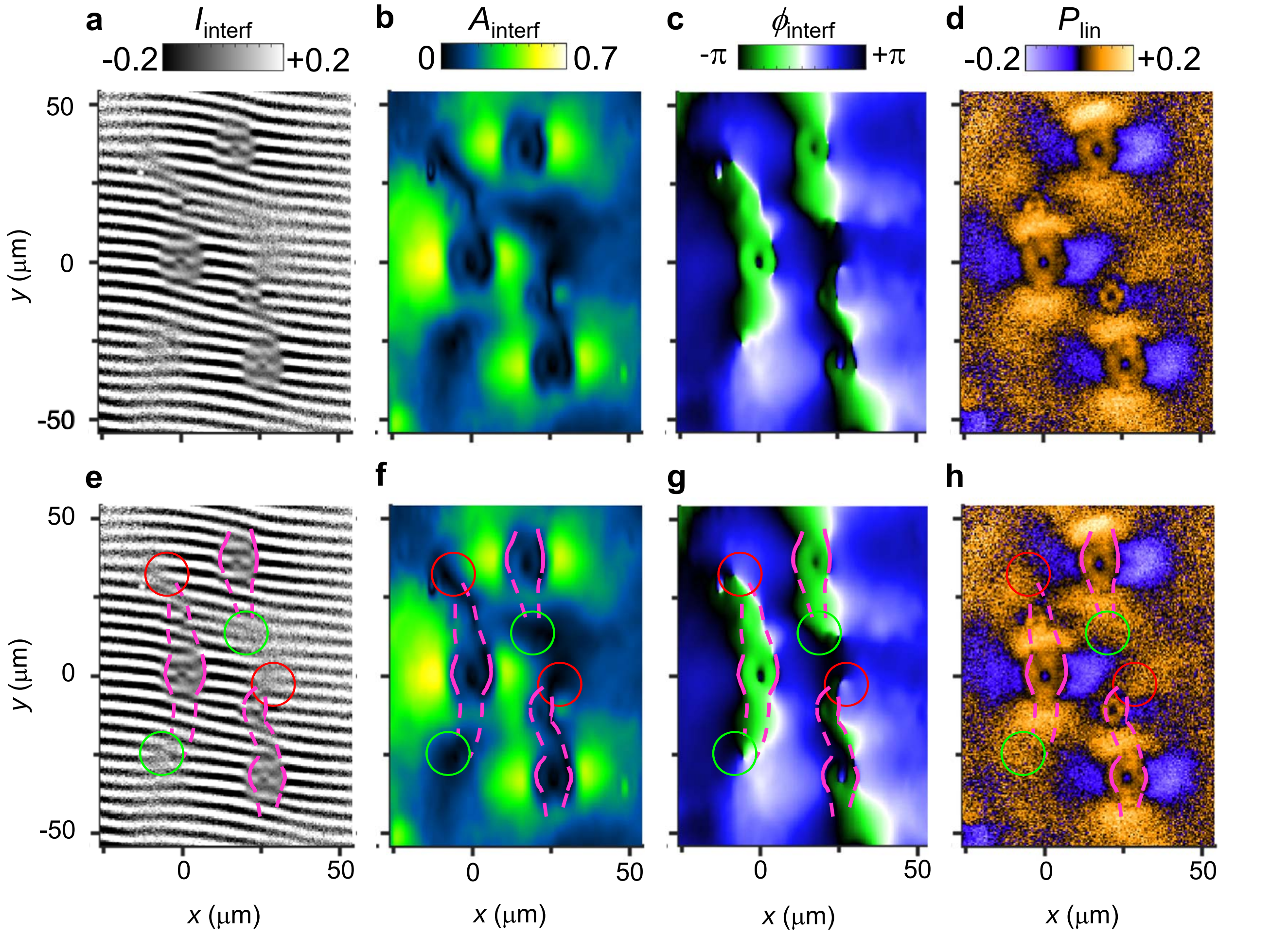

Supplementary Figure 2: **Coherence and polarization patterns of indirect excitons (IXs).** **a,e** Shift-interference pattern of IX emission, $I_{\rm interf}(x,y)$. The shift $\delta x = 2$ μm. **b,c,f,g** Amplitude, $A_{\rm interf}(x,y)$, **b,f** and phase, $\phi_{\rm interf}(x,y)$, **c,g** of interference fringes in **a,e**. **d,h** The linear polarization of IX emission, $P_{\rm linear}(x,y)$. **a-d** show the raw data without markup. In **e**, the positions of phase jumps of interference fringes are marked by magenta lines and the positions of left (right) forks of interference fringes are marked by green (red) circles. The lines are solid in the circular region around each LBS where the jumps are sharp and dashed outside these regions where the jumps are smoother. These lines and circles are copied to **f-h** to show spatial correlations in $A_{\rm interf}(x,y)$, $\phi_{\rm interf}(x,y)$, $P_{\rm linear}(x,y)$. Excitation power $P = 1.2$ mW. $T_{\rm bath} = 0.1$ K.

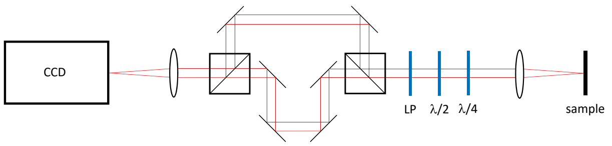

Supplementary Figure 3: **Imaging Setup.** Schematic of the shift-interferometry and polarization imaging setup.

Supplementary Note 4: Theory

We use a theoretical model which describes the appearance of the IX polarization textures and links them to spin currents carried by electrons and holes bound into bright and dark IX states. This model is based on ballistic IX transport out of the LBS origin and coherent electron and hole spin precession [2, 3]. Ballistic IX transport originates from the suppression of scattering in the condensate of IXs. Coherent electron and hole spin precession originate from the suppression of spin relaxation in the condensate of IXs. The states with different spins are split due to the splitting of linearly polarized IX states and spin-orbit interaction, which is described by the Dresselhaus Hamiltonian $H_{\rm e} = \beta_{\rm e}\left(k_x^{\rm e}\sigma_x - k_y^{\rm e}\sigma_y\right)$ for electrons and $H_{\rm h} = \beta_{\rm h}\left(k_x^{\rm h}\sigma_x + k_y^{\rm h}\sigma_y\right)$ for holes [4–6] ($\mathbf{k}_{\rm e,h}$ are electron and hole wave-vectors given by $k_{\rm e} = k_{\rm ex} m_{\rm e}/(m_{\rm e}+m_{\rm h})$, $k_{\rm h} = k_{\rm ex} m_{\rm h}/(m_{\rm e}+m_{\rm h})$, $m_{\rm e}$ and $m_{\rm h}$ are in-plane effective

masses of electron and heavy hole, respectively, $k_{\rm ex}$ is the exciton wave vector, $\beta_{\rm e,h}$ are constants, and $\sigma_{x,y}$ are Pauli matrices). In the basis of four IX states with spins $J_z = +1, -1, +2, -2$, the coherent spin dynamics in the system is governed by a model matrix Hamiltonian:

$$\hat{H} = \begin{bmatrix} E_{\rm b} & -\delta_{\rm b} & k_{\rm e}\beta_{\rm e}e^{-i\phi} & k_{\rm h}\beta_{\rm h}e^{-i\phi} \\ -\delta_{\rm b} & E_{\rm b} & k_{\rm h}\beta_{\rm h}e^{i\phi} & k_{\rm e}\beta_{\rm e}e^{i\phi} \\ k_{\rm e}\beta_{\rm e}e^{i\phi} & k_{\rm h}\beta_{\rm h}e^{-i\phi} & E_{\rm d} & -\delta_{\rm d} \\ k_{\rm h}\beta_{\rm h}e^{i\phi} & k_{\rm e}\beta_{\rm e}e^{-i\phi} & -\delta_{\rm d} & E_{\rm d} \end{bmatrix}. \quad (4)$$

where $E_{\rm b}$ and $E_{\rm d}$ are energies of bright and dark IXs in an ideal isotropic QW, $\delta_{\rm b}$ and $\delta_{\rm d}$ describe the effect of in-plane anisotropy resulting in the splitting of IX states linearly polarized along the axes of symmetry. The angle φ is measured from the x axis.

This Hamiltonian governs the dynamics of a spin density matrix,

$$\hat{\rho} = |\Psi\rangle\langle\Psi| = \begin{bmatrix} \Psi_{+1}\Psi^*_{+1} & \Psi_{+1}\Psi^*_{-1} & \Psi_{+1}\Psi^*_{+2} & \Psi_{+1}\Psi^*_{-2} \\ \Psi_{-1}\Psi^*_{+1} & \Psi_{-1}\Psi^*_{-1} & \Psi_{-1}\Psi^*_{+2} & \Psi_{-1}\Psi^*_{-2} \\ \Psi_{+2}\Psi^*_{+1} & \Psi_{+2}\Psi^*_{-1} & \Psi_{+2}\Psi^*_{+2} & \Psi_{+2}\Psi^*_{-2} \\ \Psi_{-2}\Psi^*_{+1} & \Psi_{-2}\Psi^*_{-1} & \Psi_{-2}\Psi^*_{+2} & \Psi_{-2}\Psi^*_{-2} \end{bmatrix}, \quad (5)$$

according to the quantum Liouville equation

$$i\hbar\frac{d\hat{\rho}}{dt} = [\hat{H}, \hat{\rho}]. \quad (6)$$

Here $\Psi = (\Psi_{+1}, \Psi_{-1}, \Psi_{+2}, \Psi_{-2})$ is the exciton wavefunction projected to four spin states.

The components of the Stokes vector S_1, S_2, and S_3 and the polarization degree of light emitted by IXs are given by the elements of the density matrix $\hat{\rho}$. The circular polarization degree is given by

$$\rho_{\rm c} = S_3 = (\rho_{11} - \rho_{22})/(\rho_{11} + \rho_{22}), \quad (7)$$

the linear polarization degree is given by

$$\rho_{\rm l} = S_1 = (\rho_{12} + \rho_{21})/(\rho_{11} + \rho_{22}), \quad (8)$$

and the linear polarization degree measured in the diagonal axes (also referred to as a diagonal polarization degree) is given by

$$\rho_{\rm d} = S_2 = i(\rho_{12} - \rho_{21})/(\rho_{11} + \rho_{22}). \quad (9)$$

In order to obtain the spatial distribution of Stokes vector components in the cw regime we assume that IXs propagate in radial directions from a point-like or a ring-like source. Their polarization state in a point characterized by the polar coordinates (r, ϕ) is obtained from the elements of the density matrix $\hat{\rho}(t, \phi)$ with $r = tv_{\rm ex}$ where $v_{\rm ex} = \hbar k_{\rm ex}/(m_{\rm e} + m_{\rm h})$ is IX velocity.

The initial IX state considered by this model is a ring around the LBS center where the IX gas is classical. The ring radius is taken 4 μm. In the simulations presented in Fig. 5a, 5b, and 5c, we use x-polarization as the initial condition to follow the experiment: The x-polarization is observed at $r < r_{\rm linear} = r_{\rm coh} = r_{\rm phase}$ before IXs condense and coherent precession starts, see Fig. 1e, 2c, and 2d. No simulations were performed inside this ring. For IX states on this ring, the simulations consider the classical IX energy distribution, i.e., the distribution with the density matrix $\hat{\rho}(t = 0) = \exp(-\hat{H}/{\rm k}T)$. As discussed in Supplementary Reference [2], spin-orbit interaction, exchange interaction, and the crystal field lift the degeneracy of the four exciton spin states and create preferential spin directions. Beyond this ring, the simulations consider ballistic IX transport with coherent spin precession. We use parameter values $\beta_{\rm e} = 2.7$ μeVμm, $\beta_{\rm h} = 0.92$ μeVμm, $\delta_{\rm b} = 0.5$ μeV, $\delta_{\rm d} = -13$ μeV, $E_{\rm b} - E_{\rm d} = 5$ μeV, $k_{\rm ex} = 15.4$ μm^{-1}, and $T = 0.1$ K. These parameters were chosen following earlier studies, see Supplementary Reference [2]. The details of this model are presented in [2, 3]. Improvements of the model and fitting the parameters to the measured phase shifts can be subjects of future work. In particular, a model can be made more realistic by including exciton interaction, generation, energy relaxation, and recombination.

Supplementary References

[1] Milonni, P.W., Eberly, J.H. *Lasers* (Wiley, New York, 1988).

[2] High, A.A., Hammack, A.T., Leonard, J.R., Yang, Sen, Butov, L.V., Ostatnický, T., Vladimirova, M., Kavokin, A.V., Liew, T.C.H., Campman, K.L., Gossard, A.C. Spin currents in a coherent exciton gas. *Phys. Rev. Lett.* **110**, 246403 (2013).

[3] Kavokin, A.V., Vladimirova, M., Jouault, B., Liew, T.C.H., Leonard, J.R., Butov, L.V. Ballistic spin transport in exciton gases. *Phys. Rev. B* **88**, 195309 (2013).

[4] Rashba, E.I., Sherman, E.Ya. Spin-orbital band splitting in symmetric quantum wells, *Phys. Lett. A* **129**, 175 (1988).

[5] Wu, Congjun, Mondragon-Shem, Ian. Exciton condensation with spontaneous time-reversal symmetry breaking, preprint at http://arXiv.org/abs/0809.3532v1 (2008).

[6] Luo, J.-W., Chantis, A.N., van Schilfgaarde, M., Bester, G., Zunger, A. Discovery of a Novel Linear-in-k Spin Splitting for Holes in the 2D GaAs/AlAs System, *Phys. Rev. Lett.* **104**, 066405 (2010).
